# Discrete curvature and torsion from cross-ratios

**DOI:** 10.1007/s10231-021-01065-x

**Published:** 2021-01-21

**Authors:** Christian Müller, Amir Vaxman

**Affiliations:** 1 Institute of Discrete Mathematics and Geometry, TU Wien, Wiedner Hauptstraße 8-10/104, 1040 Vienna, Austria; 2 Department of Information and Computing Sciences, Utrecht University, Princetonplein 5, De Uithof, 3584 CC Utrecht, The Netherlands

**Keywords:** Discrete curvature, Discrete torsion, Asymptotic analysis, 41A60, 52Cxx, 53A40

## Abstract

Motivated by a Möbius invariant subdivision scheme for polygons, we study a curvature notion for discrete curves where the cross-ratio plays an important role in all our key definitions. Using a particular Möbius invariant point-insertion-rule, comparable to the classical four-point-scheme, we construct circles along discrete curves. Asymptotic analysis shows that these circles defined on a sampled curve converge to the smooth curvature circles as the sampling density increases. We express our discrete torsion for space curves, which is not a Möbius invariant notion, using the cross-ratio and show its asymptotic behavior in analogy to the curvature.

## Introduction

Many topics in applied geometry like computer graphics, computer vision, and geometry processing in general, cover tasks like the acquisition and analysis of geometric data, its reconstruction, and further its manipulation and simulation. Numerically stable approximations of 3D-geometric notions play a crucial part in creating algorithms that can handle such tasks. In particular the estimation of *curvatures* of curves and surfaces is needed in these geometric algorithms [3, 10, 12]. A good understanding of estimating curvatures of curves often serves as an important first step for estimating curvatures of surfaces.

A different approach to discrete curvatures comes from discrete differential geometry [2]; the motivation behind any discretization is to apply the ideas and methods from classical differential geometry instead of “simply” discretizing equations or using classical differential calculus. Discrete curvatures defined via this approach are thus connected to a sensible notion of a curvature circle [9], a consistent definition of a Frenet-frame [5], or geometric ideas that appear in geometric knot theory [14]. Sometimes discrete definitions of “differential” notions of curves are justified to be sensible via asymptotic analysis and convergence behavior [11, 13].

In the present paper, we combine both strategies in a way. For example, we show the invariance of our discrete curvature circle with respect to Möbius transformations or characterize classes of discrete curves that are Möbius equivalent to an arc length parametrization. On the other hand, and therein lies our focus, we use asymptotic analysis to justify the definitions of our discrete notions. For example, in analogy to Sauer [13], we discretize/sample a smooth curve *s*(*t*) by constructing the inscribed polygon $s(k \varepsilon )$ with $k \in \mathbb {Z}$ as depicted in Fig. 2 (right). Using this discrete curve, we prove that our discrete curvature $\kappa _k$, which is defined at the polygon edge $k, k + 1$, is a second-order approximation of the curvature $\kappa$ of *s*, i.e., $\kappa _k = \kappa + \mathrm{O}(\varepsilon ^2)$ as $\varepsilon \rightarrow 0$ (see Theorem 2). Our definition of $\kappa _k$ will use four consecutive points as input. From our definition of the curvature circle we immediately obtain a discrete Frenet-frame in Theorem 3 and Sect. 5.2.

In our definition of the discrete curvature circle appears the cross-ratio of four points as main ingredient of its definition. The Möbius invariance of the cross-ratio thus implies the same for the curvature circle, analogous to smooth curves. The cross-ratio are also used in our definition for the torsion, a geometric quantity that is not Möbius invariant.

Our exposition starts with setting the scene in the preliminaries (Sec. 2). Then we investigate a notion of discrete curvatures notion for planar curves (Sec. 3) which we generalize to space curves in Sect. 4. In Sect. 5 we examine a discrete notion of torsion for three-dimensional curves. In Sect. 6 we consider some special cases and geometric properties of a particular ‘point-insertion-rule’ (Eqn. (3)) that plays an important rule in our definition of the discrete curvature. Finally, in Sect. 7 we perform numerical experiments to convince the robustness our discrete notions of curvature and torsion.

## Preliminaries

### Quaternions

The Hamiltonian quaternions $\mathbb {H}$ are very well suited for expressing geometry in three dimensional space and in particular for three dimensional Möbius geometry (Sec. 2.2). The quaternions constitute a skew field whose elements can be identified with $\mathbb {R}\times \mathbb {R}^3$. In this paper we write quaternions in the following way:

$$\begin{aligned} \mathbb {H}= \{[r, v] \mid r \in \mathbb {R}, v \in \mathbb {R}^3\}. \end{aligned}$$

The first component $r = \mathrm{Re}q$ of a quaternion $q = [r, v]$ is called the *real part*, and the second component $v = \mathrm{Im}q$ the *imaginary part*. Consequently, we write $\mathrm{Im}\mathbb {H}= \{q \in \mathbb {H}\mid q = [0, v],\ \text {with}\ v \in \mathbb {R}^3\}$. The addition in this notation of $\mathbb {H}$ reads $[r, v] + [s, w] = [r + s, v + w]$, while the multiplication reads $[r, v] \cdot [s, w] = [rs - \langle v, w\rangle , r w + s v + v \times w]$, where $\langle \cdot , \cdot \rangle$ is the Euclidean scalar product in $\mathbb {R}^3$ and where $\times$ is the cross product. The *conjugation* of $q = [r, v]$ is defined by ${\overline{q}} = [r, -v]$, and the square root of the real number $q {\bar{q}}$ is called *norm* of *q*, denoted by $|q| = \sqrt{q \bar{q}}$. For every $q \in \mathbb {H}\setminus \{ 0 \}$ its *inverse* is given by $q^{-1} = {\overline{q}}/|q|^2$.

Any quaternion $q \in \mathbb {H}$ can be represented via its *polar representation* $q = |q| [\cos \phi , v \sin \phi ]$ with $\Vert v\Vert = 1$ and $\phi \in [0, \pi ]$. In that case we can define the square root of *q* by $\sqrt{q} = \sqrt{|q|} [\cos \frac{\phi }{2}, v \sin \frac{\phi }{2}]$. Also when computing the square root of a complex number we will always choose the principal square root $\sqrt{z} = \sqrt{|z|} \exp (i \phi /2)$ for $z = |z| \exp (i \phi )$ with $\phi \in (-\pi , \pi ]$. The case of $z \in \mathbb {R}_{< 0}$ will not play any role in what follows (as explained in Sec. 3.1 and Sec. 4.2).

Finally, to express points and vectors in $\mathbb {R}^3$ with quaternions, we identify $\mathbb {R}^3$ with $\mathrm{Im}\mathbb {H}$ via $v \leftrightarrow [0, v]$.

### Möbius geometry

A *Möbius transformation* is a concatenation of a finite number of reflections $\sigma$ in spheres (center *c*, radius *r*); hence, $\sigma : \mathbb {R}^n \cup \{\infty \} \rightarrow \mathbb {R}^n \cup \{\infty \}$ with $\sigma (x) = (x - c)/\Vert x - c\Vert ^2 + c$, $\sigma (\infty ) = c$, $\sigma (c) = \infty$. Invariants in Möbius geometry are consequently notions and objects that stay invariant under Möbius transformations. An important example of an invariant of planar Möbius geometry is the complex cross-ratio.

### Cross-ratio

The cross-ratio is a fundamental notion in geometry, in particular Möbius geometry. For four quaternionic numbers $a, b, c, d \in \mathbb {H}$ the *cross-ratio* is defined as

$$\begin{aligned} \mathrm{cr}(a, b, c, d) := (a - b) (b - c)^{-1} (c - d)(d - a)^{-1}, \end{aligned}$$

and it is therefore a quaternion itself. The complex numbers $\mathbb {C}$ constitute a subfield in $\mathbb {H}$. In our notation $\mathbb {C}$ can be embedded in $\mathbb {H}$ as $\mathbb {C}\cong \{q \in \mathbb {H}\mid q = [r, (x, 0, 0)],\ \text {with}\ r, x \in \mathbb {R}\}$. Consequently, the cross-ratio for complex numbers can be written in the form

$$\begin{aligned} \mathrm{cr}(a, b, c, d) = \frac{(a - b) (c - d)}{(b - c)(d - a)}, \end{aligned}$$

as $\mathbb {C}$ is commutative.

It is well known that the cross-ratio of four points in $\mathbb {R}^3$ or in $\mathbb {C}$ is real if and only if the four points are concyclic (see e.g. [1]).

### Smooth curves

Our goal is to define a notion of curvature and torsion for discrete curves (Sec. 2.5). We will compare our discrete notions to those of the classical (smooth) differential geometry and as such to parametrized curves $s: \mathbb {R}\rightarrow \mathbb {R}^3$. We will always assume *s* to be sufficiently differentiable. The *curvature* $\kappa$ and *torsion* $\tau$ of *s* are given by (see e.g. [7])

1 $$\begin{aligned} \kappa = \frac{\Vert s' \times s''\Vert }{\Vert s'\Vert ^3}, \quad \text {and}\quad \tau = -\frac{\langle s' \times s'', s'''\rangle }{\Vert s' \times s''\Vert ^2}. \end{aligned}$$

The torsion vanishes if and only if the curve is planar. For a planar curve $s: \mathbb {R}\rightarrow \mathbb {R}^2$ the curvature is the oriented quantity

2 $$\begin{aligned} \kappa = \frac{\det (s', s'')}{\Vert s'\Vert ^3}. \end{aligned}$$

### Discrete curves

By a *discrete curve*, we refer to a polygonal curve in $\mathbb {R}^2$ or $\mathbb {R}^3$ which is given by its vertices via a map $\gamma : \mathbb {Z}\rightarrow \mathbb {R}^3$. For visual analogy to the notion of a smooth curve we connect for all $i \in \mathbb {Z}$ consecutive vertices $\gamma (i) \gamma (i + 1)$ by a straight line segment, which we call the *edges*. However, connecting by line segments is not crucial in this paper except for better visualizations in our illustrations. To shorten the notation we will write $\gamma _i$ for $\gamma (i)$. We call the discrete curve *planar* if it is contained in a plane, i.e., in a two dimensional affine subspace.

## Curvature of planar discrete curves

We first begin our investigation with a discrete curvature notion for *planar* curves and extend it in Sect. 4 to curves in $\mathbb {R}^3$. We identify the two dimensional plane in which our curves live with the plane of complex numbers $\mathbb {C}$. Before we proceed to the definition of the curvature (Sec. 3.2) we will consider a ‘point-insertion-rule’ in Sect. 3.1. We have also considered this point-insertion-rule in the context of a Möbius invariant subdivision method in [16].

### Point-insertion-rule in $\mathbb {C}$

The square root is not uniquely defined in our formulation (see Sec. 2.1) for negative real numbers. So we must exclude that case in the following which is not a significant restriction as this case (i.e., $\mathrm{cr}(c, a, b, d) \in \mathbb {R}_{< 0})$ corresponds to a concyclic quadrilateral *a*, *b*, *c*, *d* with *a* separated from *d* by *b* and *c* on the circumcircle. We exclude such “zigzag” quadrilaterals in the following and consider them as discrete singularities of our polygons.

Let $a, b, c, d \in \mathbb {C}$ be four pairwise distinct points. We construct a new point $f(a, b, c, d) \in \mathbb {C}$ in an, at a first glance, very unintuitive way:

3 $$\begin{aligned} f(a, b, c, d) := \frac{c (b - a) \sqrt{\mathrm{cr}(c, a, b, d)} + b (c - a)}{(b - a) \sqrt{\mathrm{cr}(c, a, b, d)} + (c - a)} \in \mathbb {C}\cup \infty . \end{aligned}$$

We will explain more about special cases and the geometric relation of *f* with respect to *a*, *b*, *c*, *d* in Sect. 6.

#### **Lemma 1**

*The newly inserted point* *f*(*a*, *b*, *c*, *d*) *fulfills*

$$\begin{aligned} \mathrm{cr}(c, a, b, f(a, b, c, d)) = -\sqrt{\mathrm{cr}(c, a, b, d)}. \end{aligned}$$

*In particular the construction of* *f* *is Möbius invariant.*

#### *Proof*

We expand the cross-ratio on the left hand side and obtain

$$\begin{aligned} \frac{(c - a) (b - f)}{(a - b) (f - c)} = -\sqrt{\mathrm{cr}(c, a, b, d)}. \end{aligned}$$

Now simple manipulations of this equation yield (3). The Möbius invariance follows immediately, as *f* can be expressed just in terms of cross-ratios. $\square$

**Fig. 1:**
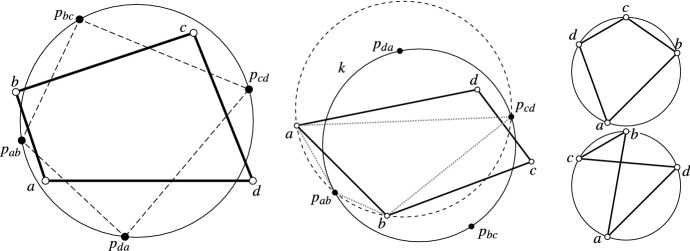
Left: A quadrilateral $a, b, c, d \in \mathbb {C}$ with its newly inserted points $p_{ab}, p_{bc}, p_{cd}, p_{da}$ which have a cross-ratio of $-1$ and therefore lie on a common circle. Center: The four points $a, p_{ab}, b, p_{cd}$ also have a cross-ratio of $-1$ and lie therefore also on a common circle *k*. Furthermore, $\mathrm{cr}(a, p_{ab}, b, p_{cd}) = -1$ implies that the pair (*a*, *b*) is separated by the pair $(p_{ab}, p_{cd})$. Consequently, *a* and *b* lie on different sides of *k*. Right: Two concyclic quadrilaterals, convex (top) and non-convex with crossing edges (bottom)

#### **Theorem 1**

*Let* $a, b, c, d \in \mathbb {C}$ *be four pairwise distinct points and consider the four new points obtained from* *f* *by cyclic permutation:*

$$\begin{aligned} p_{ab} = f(d, a, b, c),\ p_{bc} = f(a, b, c, d),\ p_{cd} = f(b, c, d, a),\ p_{da} = f(c, d, a, b). \end{aligned}$$

*Then* $p_{ab}, p_{bc}, p_{cd}, p_{da}$ *are concyclic with* $\mathrm{cr}(p_{ab}, p_{bc}, p_{cd}, p_{da}) = -1$ *(see Fig.* 1*left).*

#### *Proof*

It is a well-known fact that the cross-ratio of four points is real if and only if the four points lie on a circle. Hence, we only have to show the second part; namely,

4 $$\begin{aligned} \frac{(p_{ab} - p_{bc}) (p_{cd} - p_{da})}{(p_{bc} - p_{cd})(p_{da} - p_{ab})} = -1, \ \text {or equivalently}\ 2 p_{ab} p_{cd} + 2 p_{bc} p_{da} = (p_{ab} + p_{cd})(p_{bc} + p_{da}). \end{aligned}$$

Another well known (and readily verifiable) fact about the cross-ratio is $\mathrm{cr}(b, a, d, c) = \mathrm{cr}(a, b, c, d)$. Consequently, the cross-ratios that appear in the definition of $p_{ab}$ and $p_{cd}$ are the same as well as in $p_{bc}$ and $p_{da}$. So, let us denote the cross-ratios by $q := \mathrm{cr}(c, a, b, d) = \mathrm{cr}(a, c, d, b)$ and start to collect the terms of the second equation of (4):

$$\begin{aligned} p_{bc} p_{da}&= \frac{c (b - a) \sqrt{q} + b (c - a)}{(b - a) \sqrt{q} + (c - a)} \cdot \frac{a (d - c) \sqrt{q} + d (a - c)}{(d - c) \sqrt{q} + (a - c)} = \cdots = \\&= \frac{(1 + \sqrt{q}) (a b d - a b c - b c d + a c d)}{(1 + \sqrt{q}) (a - b - c + d)} = \frac{a b d - a b c - b c d + a c d}{a - b - c + d}. \end{aligned}$$

As $p_{ab}$ and $p_{cd}$ result from $p_{da}$ and $p_{bc}$, respectively, by a cyclic permutation of one step ($a \rightarrow b, b \rightarrow c, c \rightarrow d, d \rightarrow a$), we immediately obtain by shifting from the last identity

5 $$\begin{aligned} p_{ab} p_{cd} = \frac{a b c - b c d - a c d + a b d}{a + b - c - d}. \end{aligned}$$

Next we compute the factors of the right hand side:

$$\begin{aligned} p_{bc} + p_{da} = \frac{c (b - a) \sqrt{q} + b (c - a)}{(b - a) \sqrt{q} + (c - a)} + \frac{a (d - c) \sqrt{q} + d (a - c)}{(d - c) \sqrt{q} + (a - c)} = \ldots = \frac{2 a d - 2 b c}{a - b - c + d}, \end{aligned}$$

and again the same permutation of one shift yields

6 $$\begin{aligned} p_{ab} + p_{cd} = \frac{2 a b - 2 c d}{a + b - c - d}. \end{aligned}$$

Adding and multiplying these notions together yields Eq. (4). $\square$

Lemma 1 immediately implies the following two important consequences:

#### **Corollary 1**

(i) *If a*, *b*, *c*, *d lie on a circle such that* $\mathrm{cr}(c, a, b, d) > 0$ *(which is the case for a convex quadrilateral, i.e., non-crossing edges; see Fig.* 1*right) then the four points* $p_{ab}, p_{bc}, p_{cd}, p_{da}$ *lie on the same circle.*
(ii) *The circle given by Theorem* 1*is connected to a*, *b*, *c*, *d* *in a Möbius invariant way.*

#### **Lemma 2**

*For any four pairwise distinct points* $a, b, c, d \in \mathbb {C}$, *the harmonic conjugate of* $p_{ab}$ *with respect to* *a*, *b* *is* $p_{cd}$, *which equivalently means* $\mathrm{cr}(a, p_{ab}, b, p_{cd}) = -1$ (*see also Fig.* 1*center). Analogously, for the other quadruples we have* $\mathrm{cr}(b, p_{bc}, c, p_{da}) = -1$, $\mathrm{cr}(c, p_{cd}, d, p_{ab}) = -1$, *and* $\mathrm{cr}(d, p_{da}, a, p_{bc}) = -1$.

#### *Proof*

We show

$$\begin{aligned} \mathrm{cr}(a, p_{ab}, b, p_{cd}) = -1, \quad \text {or equivalently,}\quad 2 a b + 2 p_{ab} p_{cd} = (p_{ab} + p_{cd}) (a + b). \end{aligned}$$

The product $p_{ab} p_{cd}$ has been computed before in Eq. (5), and the sum $p_{ab} + p_{cd}$ in Eq. (6). Multiplying these terms together as written above on the right hand side concludes the proof. $\square$

#### **Corollary 2**

*Let* $a, b, c, d \in \mathbb {C}$ *be four pairwise distinct points and let* *k* *denote the circle through* $p_{ab}, p_{bc}, p_{cd}, p_{da}$. *Then either all eight points lie on the same circle* *k*, *or* *a*, *c* *lie on one side of* *k* *and* *b*, *d* *on the other side (see Fig.* 1*center).*

#### *Proof*

The two points $p_{ab}$ and $p_{cd}$ lie on the circle *k*. Suppose *a* lies outside of *k* as depicted in Fig. 1 (center). Then Lemma 2 implies that *b* lies on a circle through $a, p_{ab}, p_{cd}$, and further, $\mathrm{cr}(a, p_{ab}, b, p_{cd}) = -1$ which implies that the pair $(p_{ab}, p_{cd})$ separates the pair (*a*, *b*) (cf. [6]). Consequently, *b* lies inside *k*. The same argument then implies that *c* lies outside again and further *d* inside. $\square$

**Fig. 2:**
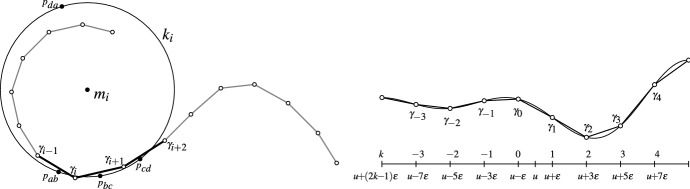
Left: A planar discrete curve $\gamma : \mathbb {Z}\rightarrow \mathbb {C}$ with the discrete curvature circle $k_i$ at edge $\gamma _i \gamma _{i + 1}$ (the points $p_{ab}$ correspond to $p_{\gamma _{i - 1} \gamma _{i}}$ etc). Right: Sampling a smooth curve $s: \mathbb {R}\rightarrow \mathbb {R}^2$ at $u + (2 k - 1) \varepsilon$ to obtain the discrete curve $\gamma : \mathbb {Z}\rightarrow \mathbb {R}^2$ with $\gamma _k = s(u + (2 k - 1) \varepsilon )$. For our asymptotic analysis we let the real number $\varepsilon$ go to zero

### Curvature for planar curves

Let us consider the planar discrete curve $\gamma : \mathbb {Z}\rightarrow \mathbb {C}$ as illustrated in Fig. 2 (left). We assume that any four consecutive vertices of the curve are pairwise distinct. Then, Thm. 1 guarantees the existence of a circle $k_i$ passing through $f(\gamma _{i - 1}, \gamma _{i}, \gamma _{i + 1}, \gamma _{i + 2})$, $f(\gamma _{i}, \gamma _{i + 1}, \gamma _{i + 2}, \gamma _{i - 1})$, $f(\gamma _{i + 1}, \gamma _{i + 2}, \gamma _{i - 1}, \gamma _{i})$, $f(\gamma _{i + 2}, \gamma _{i - 1}, \gamma _{i}, \gamma _{i + 1})$. We use this circle $k_i$ in the following definition of our discrete curvature.

#### **Definition 1**

Let $\gamma : \mathbb {Z}\rightarrow \mathbb {C}$ be a planar discrete curve. We call the circle $k_i$ *(discrete) curvature circle at the edge* $\gamma _{i}\gamma _{i + 1}$, the inverse of its radius *(discrete) curvature* $\kappa _i$ *at the edge* $\gamma _{i}\gamma _{i + 1}$, and its center $m_i$ *(discrete) curvature center*. For an illustration see Fig. 2 (left).

A ‘good’ discrete definition ‘mimics’ its smooth counterparts. Along these lines we note that our discrete curvature circle is Möbius invariant (Corollary 1 (ii)) as in the smooth case. Furthermore, the curvature circle of a discrete curve with vertices on a circle—we could call it a discrete circle—is the circumcircle itself, as expected. And Corollary 2 implies that the curvature circle separates the first and the last point of the four points that are involved in its definition (see Fig. 2 left). This resembles the local behavior of smooth curves at non-vertex points where the curvature circle locally separates the curve into an ‘inner’ and an ‘outer’ curve.

**Fig. 3:**
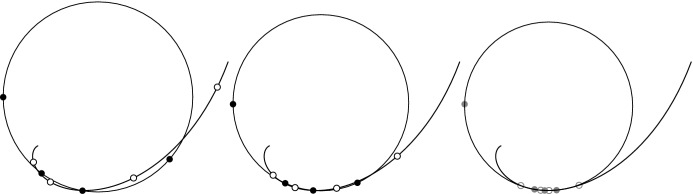
The white points mark four sampled points which move closer and closer to a common point from left to right. The associated discrete curvature circle (exactly passing through the black points) converges at the same time to the smooth curvature circle

In the following we continue our justification of this definition of the discrete curvature circle using *asymptotic analysis*. We will show that the discrete curvature circle (its radius and center) of a sampled curve *s* converges to the smooth curvature circle as the sampling gets denser and denser. For an illustration of the setting of the following theorem, see Figs. 2 and 3.

#### **Theorem 2**

*Let* $s: \mathbb {R}\rightarrow \mathbb {C}$ *be a sufficiently smooth planar curve and let* $u, \varepsilon \in \mathbb {R}$. *Further let* $\gamma : \mathbb {Z}\rightarrow \mathbb {C}$ *be the planar discrete curve that samples the smooth curve* *s* *in the following way:*

$$\begin{aligned} \gamma _k = \gamma (k) := s(u + (2 k - 1)\varepsilon ) \qquad k \in \mathbb {Z}. \end{aligned}$$

*Then the* *discrete* *curvature* $\kappa _0$ *of* $\gamma$ *at the edge* $\gamma _0 \gamma _1$ *is a second-order approximation of the* *smooth* *curvature* $\kappa$ *of* *s* *at* *u*:

7 $$\begin{aligned} \kappa _0 = |\kappa (u)| + \mathrm{O}(\varepsilon ^2). \end{aligned}$$

*The center* $m_0$ *of the* *discrete* *curvature circle* $k_0$ *of* $\gamma$ *converges to the center of the* *smooth* *curvature circle of* *s* *at the same rate,*

8 $$\begin{aligned} m_0 = s(u) + \frac{1}{\kappa (u)} N(u) + \mathrm{O}(\varepsilon ^2), \end{aligned}$$

*where* *N* *denotes the unit normal vector along* *s*. *Furthermore,* $p_{\gamma _0 \gamma _1} = f(\gamma _{-1}, \gamma _{0}, \gamma _{1}, \gamma _{2})$ *is even a third-order approximation of* *s*(*u*), *i.e.,*

9 $$\begin{aligned} p_{\gamma _0 \gamma _1} = s(u) + \mathrm{O}(\varepsilon ^3). \end{aligned}$$

Before we give a proof of this theorem,we need a couple of preparatory lemmas. We consider without loss of generality the approximation point at $u = 0$. To study the asymptotic behavior of the curvature notions based on our sampled curve, we need its Taylor expansion at 0:

$$\begin{aligned} s(u) = s(0) + u s'(0) + \frac{u^2}{2} s''(0) + \frac{u^3}{6} s'''(0) + \mathrm{O}(u^4). \end{aligned}$$

For the sake of brevity we will just write *s* instead of *s*(0), $s'$ instead of $s'(0)$, etc. And until the end of this section we will use the following abbreviations for those four points on which the curvature circle depends:

10 $$\begin{aligned} a := \gamma _{-1} = s(-3 \varepsilon ), \quad b := \gamma _0 = s(-\varepsilon ), \quad c := \gamma _1 = s(\varepsilon ), \quad d := \gamma _2 = s(3 \varepsilon ). \end{aligned}$$

We will very frequently encounter rational functions depending on $\varepsilon$ for which we need its Taylor expansion. So at first, we prepare a general technical lemma that can easily be verified.

#### **Lemma 3**

*Let* $x_i, y_i \in \mathbb {C}$ *with* $y_0 \ne 0$, *then*

$$\begin{aligned} \frac{\sum _{k = 0}^2 x_i \varepsilon ^i}{\sum _{k = 0}^2 y_i \varepsilon ^i} = \frac{x_0}{y_0} + \frac{x_1 y_0 - x_0 y_1}{y_0^2} \varepsilon + \frac{x_2 y_0^2 - x_1 y_0 y_1 + x_0 y_1^2 - x_0 y_0 y_2}{y_0^3} \varepsilon ^2 + \mathrm{O}(\varepsilon ^3). \end{aligned}$$

Our first task is to compute the Taylor expansion of the cross-ratio which appears in the ‘inserting’ construction (3). The following formula illustrates also the close connection between the cross-ratio and the *Schwarzian derivative* of *s* which reads $\frac{2 s' s''' - 3 f''^2}{2 f'^2}$, cf. [4].

#### **Lemma 4**

*Let* *a*, *b*, *c*, *d* *be the four consecutive points of the sampled curve as defined in* (10). *Then*

$$\begin{aligned} \mathrm{cr}(c, a, b, d) = 4 + \frac{12 s''^2 - 8 s' s'''}{s'^2} \varepsilon ^2 + \mathrm{O}(\varepsilon ^4), \ \ \mathrm{cr}(b, d, a, c) = \frac{4}{3} + \frac{-12 s''^2 + 8 s' s'''}{9 s'^2} \varepsilon ^2 + \mathrm{O}(\varepsilon ^4). \end{aligned}$$

#### *Proof*

We start by computing the factors of

$$\begin{aligned} \mathrm{cr}(c, a, b, d) = \frac{(c - a) (b - d)}{(a - b) (d - c)} \end{aligned}$$

in terms of the Taylor expansion:

$$\begin{aligned} c - a&= s(\varepsilon ) - s(-3 \varepsilon ) = \varepsilon s' + \frac{\varepsilon ^2}{2} s'' + \frac{\varepsilon ^3}{6} s''' + \mathrm{O}(\varepsilon ^4) - \big (-3 \varepsilon s' + \frac{9 \varepsilon ^2}{2} s'' - \frac{9 \varepsilon ^3}{2} s''' + \mathrm{O}(\varepsilon ^4)\big ) \\&= 4 \varepsilon s' - 4 \varepsilon ^2 s'' + \frac{14}{3} \varepsilon ^3 s''' + \mathrm{O}(\varepsilon ^4). \end{aligned}$$

And similarly we obtain

11 $$\begin{aligned} a - b&= -2 \varepsilon s' + 4 \varepsilon ^2 s'' - \frac{13}{3} \varepsilon ^3 s''' + \mathrm{O}(\varepsilon ^4), \\ \nonumber b - d&= -4 \varepsilon s' - 4 \varepsilon ^2 s'' - \frac{14}{3} \varepsilon ^3 s''' + \mathrm{O}(\varepsilon ^4), \\ \nonumber d - c&= 2 \varepsilon s' + 4 \varepsilon ^2 s'' + \frac{13}{3} \varepsilon ^3 s''' + \mathrm{O}(\varepsilon ^4). \end{aligned}$$

Now the numerator of the cross-ratio becomes

$$\begin{aligned} (c - a) (b - d) = -16 s'^2 \varepsilon ^2 + \Big ( 16 s''^2 - \frac{112 s' s'''}{3} \Big ) \varepsilon ^4 + \mathrm{O}(\varepsilon ^6), \end{aligned}$$

while the denominator becomes

$$\begin{aligned} (a - b) (d - c) = -4 s'^2 \varepsilon ^2 + \Big ( 16 s''^2 - \frac{52 s' s'''}{3} \Big ) \varepsilon ^4 + \mathrm{O}(\varepsilon ^6). \end{aligned}$$

Consequently, after canceling $-4 \varepsilon ^2$ the cross-ratio reads

$$\begin{aligned} \mathrm{cr}(c, a, b, d) = \frac{4 s'^2 - (4 s''^2 - \frac{28}{3} s' s''') \varepsilon ^2 + \mathrm{O}(\varepsilon ^4)}{s'^2 - (4 s''^2 - \frac{13}{3} s' s''') \varepsilon ^2 + \mathrm{O}(\varepsilon ^4)}, \end{aligned}$$

which, using Lemma 3, simplifies to

$$\begin{aligned} \mathrm{cr}(c, a, b, d) = 4 + \frac{12 s''^2 - 8 s' s'''}{s'^2} \varepsilon ^2 + \mathrm{O}(\varepsilon ^4), \end{aligned}$$

the Taylor expansion of the first cross-ratio. The computations for the second one is similar. $\square$

#### **Lemma 5**

*Let* *a*, *b*, *c*, *d* *be as in Lemma* 4. *Then*

$$\begin{aligned} \sqrt{\mathrm{cr}(c, a, b, d)} = 2 + \frac{3 s''^2 - 2 s' s'''}{s'^2} \varepsilon ^2 + \mathrm{O}(\varepsilon ^3). \end{aligned}$$

#### *Proof*

This equation follows immediately from the general Taylor expansion for

$$\begin{aligned} \sqrt{x_0 + x_2 \varepsilon ^2 + \mathrm{O}(\varepsilon ^3)} = \sqrt{x_0} + \frac{x_2}{2 \sqrt{x_0}} \varepsilon ^2 + \mathrm{O}(\varepsilon ^3), \end{aligned}$$

and from Lemma 4. $\square$

Now we are in the position to show the important lemma that guarantees that $p_{\gamma _0 \gamma _1} = p_{bc}$ is a third-order approximation of *s*.

#### **Lemma 6**

*Let* *a*, *b*, *c*, *d* *be as in Lemma* 4. *Then*

$$\begin{aligned} p_{bc} = s + \mathrm{O}(\varepsilon ^3). \end{aligned}$$

#### *Proof*

We have to compute

$$\begin{aligned} p_{bc} = f(a, b, c, d) = \frac{c (b - a) \sqrt{\mathrm{cr}(c, a, b, d)} + b (c - a)}{(b - a) \sqrt{\mathrm{cr}(c, a, b, d)} + (c - a)}, \end{aligned}$$

and start with its components:

$$\begin{aligned} c (b - a)&\overset{(11)}{=} \big ( s + \varepsilon s' + \frac{\varepsilon ^2}{2} s'' + \frac{\varepsilon ^3}{6} + \mathrm{O}(\varepsilon ^4) \big ) \big ( 2 \varepsilon s' - 4 \varepsilon ^2 s'' + \frac{13}{3} \varepsilon ^3 s''' + \mathrm{O}(\varepsilon ^4) \big ) \\&= 2 \varepsilon s s' + (2 s'^2 - 4 s s'') \varepsilon ^2 + \left(\frac{13}{3} s' s''' - 3 s' s'' \right) \varepsilon ^3 + \mathrm{O}(\varepsilon ^4), \end{aligned}$$

and analogously

$$\begin{aligned} b (c - a) = 4 s s' \varepsilon - 4 (s'^2 + s s'') \varepsilon ^2 + \left(6 s' s'' + \frac{14 s s'''}{3}\right) \varepsilon ^3 + \mathrm{O}(\varepsilon ^4). \end{aligned}$$

Putting numerator and denominator together also using Lemma 5 we obtain

$$\begin{aligned} p_{bc} = \frac{8 s s' \varepsilon - 12 s s'' \varepsilon ^2 + \big ( \frac{6 s''^2}{s'} + \frac{28 s'''}{3} \big ) s \varepsilon ^3 + \mathrm{O}(\varepsilon ^4)}{8 s' \varepsilon - 12 s'' \varepsilon ^2 + \big ( \frac{6 s''^2}{s'} + \frac{28 s'''}{3} \big ) \varepsilon ^3 + \mathrm{O}(\varepsilon ^4)}, \end{aligned}$$

and after canceling $\varepsilon$, applying Lemma 3 concludes the proof. $\square$

Note that Lemma 6 proves Eq. (9) in Theorem 2 which says that $= p_{bc}$ converges to *s* at third-order. The following lemma can be verified analogously to Lemma 6.

#### **Lemma 7**

*Let* *a*, *b*, *c*, *d* *be as in* (10). *Then*

$$\begin{aligned} p_{ab}&= s - \sqrt{3} s' \varepsilon + \frac{3 s''}{2} \varepsilon ^2 + \mathrm{O}(\varepsilon ^3), \\ p_{cd}&= s + \sqrt{3} s' \varepsilon + \frac{3 s''}{2} \varepsilon ^2 + \mathrm{O}(\varepsilon ^3), \\ p_{da}&= s - \frac{2 s'^2}{s''} + \Big ( 5 s'' - \frac{20 s' s'''}{3 s''} \Big ) \varepsilon ^2 + \mathrm{O}(\varepsilon ^3). \end{aligned}$$

Lemma 7 implies that $p_{da} = p_{\gamma _{i + 2} \gamma _{i - 1}}$ is a second-order approximation of $s - \frac{2 s'^2}{s''}$. This point $s - \frac{2 s'^2}{s''}$ has an interesting geometric interpretation which we detail in Proposition 1.

After putting these preparatory lemmas in place we can finally turn to the proof of our result on the limit of the curvature circle.

#### *Proof of Theorem 2*

Let us first compute the center $m_0$ of the discrete curvature circle $k_0$. Generally, the circumcenter of a triangle $a, b, c \in \mathbb {C}^2$ is given by

$$\begin{aligned} \frac{ a (\Vert b\Vert ^2 - \Vert c\Vert ^2) + b (\Vert c\Vert ^2 - \Vert a\Vert ^2) + c (\Vert a\Vert ^2 - \Vert b\Vert ^2) }{ (a - c) (\overline{b - c}) - (\overline{a - c}) (b - c) }. \end{aligned}$$

In our case we want to compute the circumcenter of the four concyclic points $p_{ab},$ $p_{bc},$ $p_{cd},$ $p_{da}$ from which we choose the three points $p_{bc}, p_{cd}, p_{da}$ to insert them in the formula above. Let us start with the denominator ${\mathscr {D}}$:

$$\begin{aligned}&{{\mathscr {D}} =}{ (p_{da} - p_{cd}) (\overline{p_{bc} - p_{cd}}) - (\overline{p_{da} - p_{cd}}) (p_{bc} - p_{cd}) }\\&\quad {=}{ \big (-\frac{2 s'^2}{s''} - \sqrt{3} s' \varepsilon + \mathrm{O}(\varepsilon ^2)\big ) \big (-\sqrt{3} {\bar{s}}' \varepsilon - \frac{3 \bar{s}''}{2} \varepsilon ^2 + \mathrm{O}(\varepsilon ^3)\big ) - \overline{(\ldots )}(\ldots ) }\\&\quad {=}{ \frac{2 \sqrt{3} |s'|^2 (s' {\bar{s}}'' - {\bar{s}}' s'')}{|s''|^2} \varepsilon + \frac{3 (s' {\bar{s}}'' - {\bar{s}}' s'') (s' \bar{s}'' + {\bar{s}}' s'')}{|s''|^2} \varepsilon ^2 + \mathrm{O}(\varepsilon ^3). } \end{aligned}$$

We compute the numerator ${\mathcal {N}}$ in the same way. After a lengthy computation we get

$$\begin{aligned}&{{\mathcal {N}} =}{ \frac{4 \sqrt{3} |s'|^2 (|s'|^2 s' - \frac{1}{2} s (s'' {\bar{s}}' - {\bar{s}}'' s'))}{|s''|^2} \varepsilon + \frac{6 (s' {\bar{s}}'' \!+\! {\bar{s}}' s'') (|s'|^2 s' - \frac{1}{2} s (s'' {\bar{s}}' - \bar{s}'' s'))}{|s''|^2} \varepsilon ^2 }\\&\quad {+}{\mathrm{O}(\varepsilon ^3).} \end{aligned}$$

Now, Lemma 3 yields for the center $m_0$ of the discrete curvature circle $k_0$

12 $$\begin{aligned} m_0 = \frac{{\mathcal {N}}}{{\mathscr {D}}} = s + \frac{2 s'^2 {\bar{s}}'}{s' {\bar{s}}'' - {\bar{s}}' s''} + \mathrm{O}(\varepsilon ^2). \end{aligned}$$

We need to relate the discrete curvature circle to its smooth counterpart. In order to do that, we rewrite the curvature (2) in terms of complex functions: The determinant of a matrix consisting of two column vectors $a, b \in \mathbb {R}^2$ is the same as $\frac{i}{2} (a {\bar{b}} - {\bar{a}} b)$ when *a* and *b* are expressed as complex numbers. Consequently, the curvature for a curve $s: \mathbb {R}\rightarrow \mathbb {C}$ and its unit normal vector *N* can be written in the form

13 $$\begin{aligned} \kappa = \frac{i (s' {\bar{s}}'' - {\bar{s}}' s'')}{2 |s'|^3}, \quad \text {and}\quad N = i \frac{s'}{|s'|}, \end{aligned}$$

as multiplication with *i* corresponds to a rotation about the angle $\pi /2$. So, we use these notions to rewrite (12):

$$\begin{aligned} m_0 = s + \frac{|s'|^2 s' i}{\frac{i}{2}(s' {\bar{s}}'' - {\bar{s}}' s'')} + \mathrm{O}(\varepsilon ^2) = s + \frac{|s'|^3}{\det (s', s'')} i \frac{s'}{|s'|} + \mathrm{O}(\varepsilon ^2) = s + \frac{1}{\kappa } N + \mathrm{O}(\varepsilon ^2). \end{aligned}$$

Consequently, the distance between the center $m_0$ of the discrete curvature circle $k_0$ and the center $s + \frac{1}{\kappa } N$ of the smooth curvature circle is of magnitude $\mathrm{O}(\varepsilon ^2)$.

Let us now compute the radius of the discrete curvature circle $k_0$ by computing the distance of its center $m_0$ to a point on the circle, e.g., $p_{bc}$:

$$\begin{aligned} \frac{1}{\kappa _0} = |p_{bc} - m_0| = \Big |\underbrace{p_{bc} - \big (s}_{\mathrm{O}(\varepsilon ^3)} + \frac{1}{\kappa } N \big ) + \underbrace{\big (s + \frac{1}{\kappa } N \big ) - m_0}_{\mathrm{O}(\varepsilon ^2)} \Big | = \Big |\frac{1}{\kappa } N + \mathrm{O}(\varepsilon ^2)\Big | = \frac{1}{|\kappa |} + \mathrm{O}(\varepsilon ^2), \end{aligned}$$

which implies Eq. (7). Equation (9) follows from Lemma 6. $\square$

**Fig. 4:**
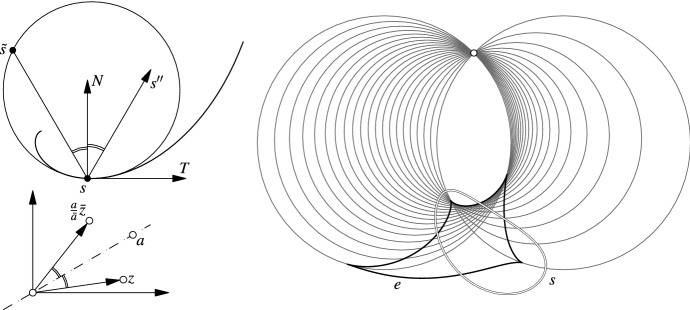
Top-left: Smooth curve *s* with curvature circle. The point ${\tilde{s}} = s - \frac{2 s'^2}{s''}$ on the curvature circle is Möbius invariantly connected to the parametrization of the curve. The vectors ${\tilde{s}} - s$ and $s''$ are symmetric, up to length, with respect to the normal vector *N*. Bottom-left: Reflection. A complex point *z* gets reflected to $\frac{a}{{\bar{a}}} {\bar{z}}$ along an axis through the origin and *a*. Right: Illustration of a curve *s* together with a family of circles which are orthogonal to *s* and which pass through ${\tilde{s}}$. All these circles pass through a fixed point which implies that the parametrization *s* is Möbius equivalent to an arc-length parametrization ${\hat{s}}$. The circles envelope the Möbius transformation of the evolute *e* of ${\hat{s}}$

In Lemma 7 we saw that the point $p_{da} = p_{\gamma _{i + 2} \gamma _{i - 1}}$ is a second-order approximation of $s(u) - \frac{2 s'^2(u)}{s''(u)}$. In the following proposition we study the geometric meaning of that special point.

#### **Proposition 1**

*Let* $s: \mathbb {R}\rightarrow \mathbb {C}$ *be a smooth curve. Then for all* $u \in \mathbb {R}$ *the point*

$$\begin{aligned} {\tilde{s}}(u) := s(u) - \frac{2 s'^2(u)}{s''(u)} \end{aligned}$$

*is a point on the curvature circle at* *s*(*u*) *(see Fig.* 4*top-left). The curve* ${\tilde{s}}$ *is Möbius-invariantly connected to the* *parametrization* *of* *s*. *Furthermore, the normal vector* *N* *of* *s* *is the angle bisector of* ${\tilde{s}} - s$ *and the second derivative vector* $s''$ *(see Fig.* 4*top-left).*

#### *Proof*

To show that ${\tilde{s}}$ lies on the curvature circle we show $\big |{\tilde{s}} - (s + \frac{1}{\kappa } N) \big | = \frac{1}{|\kappa |}$:

$$\begin{aligned} \Big |{\tilde{s}} - \Big (s + \frac{1}{\kappa } N\Big ) \Big | =&\Big |\frac{2 s'^2}{s''} + \frac{1}{\kappa } N\Big | = \Big |\frac{2 s'^2}{s''} + \frac{|s'|^3}{\frac{i}{2} (s' {\bar{s}}'' - {\bar{s}}' s'')} i \frac{s'}{|s'|} \Big | \\ =&\Big |\frac{2 s'^3 {\bar{s}}'' - 2 s'^2 {\bar{s}}' s'' + 2 s'^2 {\bar{s}}' s''}{s'' (s' {\bar{s}}'' - {\bar{s}}' s'')} \Big | = \frac{|\bar{s}''|}{|s''|} \frac{|s'|^3}{|\frac{i}{2} (s' {\bar{s}}'' - {\bar{s}}' s'')|} = \frac{1}{|\kappa |}. \end{aligned}$$

Next we show the Möbius invariant property of ${\tilde{s}}$. For that, let *M* be a Möbius transformation. We have to show

$$\begin{aligned} M \circ {\tilde{s}} = M \circ s - \frac{2 (M \circ s)'^2}{(M \circ s)''}. \end{aligned}$$

This equation holds trivially for translations, rotations and scalings. So the only thing left to show is that it is also true for inversions $M(z) = 1/z$. We start with the right hand side:

$$\begin{aligned} \frac{1}{s} - \frac{2 (\frac{1}{s})'^2}{(\frac{1}{s})''} = \frac{1}{s} + \frac{2 \frac{s'^2}{s^4}}{\frac{s'' s^2 - 2 s s'^2}{s^4}} = \frac{s''}{s'' s - 2 s'^2} = \frac{1}{s - \frac{2 s'^2}{s''}} = \frac{1}{{\tilde{s}}} = M \circ {\tilde{s}}. \end{aligned}$$

Now we show the symmetry property. We have to show that $\frac{{\tilde{s}} - s}{|{\tilde{s}} - s|}$ gets reflected to $\frac{s''}{|s''|}$ at the symmetry axis *N*. The reflection of a complex number *z* on an axis with direction *a* (see Fig. 4 bottom-left) is expressible in complex numbers by $\frac{a}{{\bar{a}}} {\bar{z}}$. So we have to show

$$\begin{aligned} \frac{N}{{\overline{N}}} \overline{\frac{{\tilde{s}} - s}{|{\tilde{s}} - s|}} = \frac{s''}{|s''|}. \end{aligned}$$

This equation is equivalent to

$$\begin{aligned} \frac{{-}i s'}{-i {\bar{s}}'} \frac{-\frac{2 s'^2}{s''}}{|\frac{2 s'^2}{s''}|} = \frac{s''}{|s''|} \quad \Leftrightarrow \quad \frac{s'}{{\bar{s}}'} \frac{{\bar{s}}'^2 |s''|}{{\bar{s}}'' |s'^2|} = \frac{s''}{|s''|} \quad \Leftrightarrow \quad \frac{s'}{{\bar{s}}'} \frac{{\bar{s}}'^2}{\bar{s}'' s' {\bar{s}}'} = \frac{s''}{s'' {\bar{s}}''}, \end{aligned}$$

which is true and therefore implies the symmetry property. $\square$

#### *Remark 1*

If *s* is parametrized proportionally to arc length, then $s \tilde{s}$ is a diameter of the curvature circle.

#### **Corollary 3**

*A parametrized curve is Möbius equivalent to an arc-length parametrized curve if and only if for all* $u \in \mathbb {R}$ *the circles orthogonal to the curvature circle and passing through* *s* *and* ${\tilde{s}}$ *intersect in one common point (see Fig.* 4*right).*

The commonly used characterization of arclength parametrizations of discrete curves is by a polygon with constant edgelengths. However, Corollary 3 implies an immediate alternative:

#### **Definition 2**

We call a discrete curve *parametrized proportionally to arclength* if $p_{\gamma _i \gamma _{i + 1}}$ and $p_{\gamma _{i + 2} \gamma _{i - 1}}$ are opposite points on the discrete curvature circle.

#### **Theorem 3**

*The unit tangent vector* $T_i$ *and unit normal vector* $N_i$ *of the discrete curvature circle* $k_i$ *at* $p_{\gamma _i \gamma _{i + 1}}$ *are second-order approximations of the unit tangent vector* *T* *and unit normal vector* *N* *of the smooth curve (after appropriate orientation), i.e.,*

$$\begin{aligned} T_i = T + \mathrm{O}(\varepsilon ^2) \quad \text {and}\quad N_i = N + \mathrm{O}(\varepsilon ^2). \end{aligned}$$

#### *Proof*

The approximation quality of *T* and *N* is the same so we just have to prove it for one of them:

$$\begin{aligned} N_i = \frac{p_{\gamma _i \gamma _{i + 1}} - m_i}{|p_{\gamma _i \gamma _{i + 1}} - m_i|} = \frac{s + \mathrm{O}(\varepsilon ^3) - \big (s - \frac{1}{\kappa } N + \mathrm{O}(\varepsilon ^2)\big )}{|s + \mathrm{O}(\varepsilon ^3) - \big (s - \frac{1}{\kappa } N + \mathrm{O}(\varepsilon ^2)\big )|} = \frac{|\kappa |}{\kappa } N + \mathrm{O}(\varepsilon ^2). \end{aligned}$$

After appropriate orientation *N* and $N_i$ differ only about $\mathrm{O}(\varepsilon ^2)$. $\square$

## Curvature for three dimensional curves

Before we generalize discrete curvature from discrete planar curves to space curves we need some more results on the quaternionic cross-ratio for points in three dimensional space. We will use the imaginary quaternions $\mathrm{Im}\mathbb {H}$ to describe points in three dimensional space $\mathbb {R}^3$ (see Sec. 2.1).

### Cross-ratio and geometry

The authors used the quaternionic algebra and the cross-ratio extensively in [15, 16] for applications in regular mesh design and for Möbius invariant subdivision algorithms. The results of this paragraph can also be found there. To prove some technical Lemmas we first consider the following geometric property, which can easily be verified.

#### **Lemma 8**

*Let* $a, b, c \in \mathbb {R}^n$ *be three points. Then* $(a - b) \Vert a - c\Vert ^2 - (a - c) \Vert a - b\Vert ^2$ *is the direction of the tangent of the circumcircle to the triangle* *abc* *at* *a*.

#### **Definition 3**

Let $a, b, c \in \mathrm{Im}\mathbb {H}$ be pairwise distinct points. Then we call the imaginary quaternion

$$\begin{aligned} t[a, b, c] := (a - b)^{-1} + (b - c)^{-1}, \end{aligned}$$

*corner tangent*.

Note that the identity $a^{-1} + b^{-1} = a^{-1} (a + b) b^{-1}$ immediately implies

14 $$\begin{aligned} t[a, b, c] = (a - b)^{-1} (a - c) (b - c)^{-1}. \end{aligned}$$

**Fig. 5:**
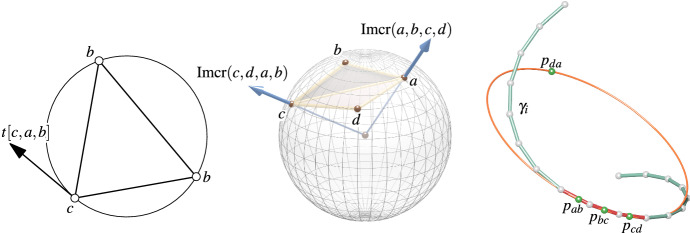
Left: The corner tangent *t*[*c*, *a*, *b*] is a vector in tangential contact with the circumcircle of a triangle (*abc*) at *a*. Center: Circumsphere of *a*, *b*, *c*, *d*. The imaginary part of the cross-ratio $\mathrm{cr}(a, b, c, d)$ is a vector that is orthogonal to the circumsphere at *a*. Right: A discrete space curve with a curvature circle. The four points $p_{ab}, p_{bc}, p_{cd}, p_{da}$ are concyclic also in the 3-space case

#### **Lemma 9**

*Consider the circumcircle of* $a, b, c \in \mathbb {H}$, *oriented according to this defining triangle. Then the vector* *t*[*c*, *a*, *b*], *placed at* *a*, *is in oriented tangential contact with the circle (see Fig.* 5*left).*

#### *Proof*

Note that $q\! \in \! \mathrm{Im}\mathbb {H}$ implies $q^{-1}\!\! =\! -q/|q|^2$. Using the definition of the corner tangent yields

$$\begin{aligned} t[c, a, b] = (c - a)^{-1} + (a - b)^{-1} = -(c - a)/|c - a|^2 - (a - b)/|a - b|^2. \end{aligned}$$

Consequently, Lemma 8 concludes the proof. $\square$

#### **Lemma 10**

*Let* $a, b, c, d \in \mathrm{Im}\mathbb {H}$ *be four points not lying on a common circle. Then, the imaginary part of the cross-ratio is the normal of the circumsphere (or plane) at* *a*, *i.e., for a* *proper* *circumsphere with center* *m*, *we have* $\mathrm{Im}\mathrm{cr}(a, b, c, d) \parallel (m - a)$ *(see Fig.* 5*center).*

#### *Proof*

We compute the cross-ratio in terms of corner tangents abbreviated by $t_1 = t[c, a, b]$ and $t_2 = t[d, a, c]$:

$$\begin{aligned} \mathrm{cr}(a, b, c, d)&= (a - b)^{{-1}}\, (b - c)^{-1}\, (c - d)^{{-1}}\, (d - a)^{-1} \\&= (a - b)^{{-1}}\, (b - c)^{-1} [(a - c)^{{-1}}\, (a - c)^{-1}] (c - d)^{{-1}}\, (d - a)^{-1}\\&= [(a - c)^{-1}\, (b - c)^{{-1}}\, (a - b)^{-1}]^{-1} [(a - c)^{-1}\, (c - d)^{{-1}}\, (d - a)^{-1}]\\&= [(a - c)^{-1}\, (b - c)^{{-1}}\, (b - a)^{-1}]^{-1} [(a - c)^{-1}\, (d - c)^{{-1}}\, (d - a)^{-1}] \\&\overset{(14)}{=} t_1^{-1}\, t_2^{{-1}}. \end{aligned}$$

Since $t_1$ and $t_2$ are both imaginary we can write the cross-ratio as

$$\begin{aligned} \mathrm{cr}(a, b, c, d) = [ \langle t_1^{-1}, t_2^{{-1}}\rangle , -t_1^{-1} \times t_2^{{-1}} ]. \end{aligned}$$

Lemma 9 implies that $t_1^{-1}$ and $t_2$ are tangent vectors to the circumcircles of the triangles (*abc*) and (*cda*), respectively, both at *a*. Consequently, the imaginary part of the above cross-ratio is the cross product of tangent vectors to circles on the circumsphere of *a*, *b*, *c*, *d* at *a*, hence orthogonal to the tangent plane of the circumsphere at *a*. $\square$

#### **Proposition 2**

*Let* $a, b, c, d \in \mathrm{Im}\mathbb {H}$ *be four non-concyclic points with* $\mathrm{cr}(a, b, c, d) = [r, v]$. *Further, let* $f \in \mathbb {H}$ *be the quaternion that solves*

$$\begin{aligned} \mathrm{cr}(a, b, c, f) = [\lambda r, \mu v], \end{aligned}$$

*for some* $\lambda , \mu \in \mathbb {R}$. *Then* $f \in \mathrm{Im}\mathbb {H}$, *i.e.,* *f* *is an imaginary quaternion representing a point in* $\mathbb {R}^3$. *Furthermore,* *f* *lies on the circumsphere of* *a*, *b*, *c*, *d*. *In particular* $f(\lambda , \mu )$ *is a parametrization of the circumsphere.*

#### *Proof*

The two occurring cross-ratios can be expressed as (see the proof of Lemma 10)

$$\begin{aligned} \mathrm{cr}(a, b, c, d) = t_1 \cdot t_2, \quad \text {and}\quad \mathrm{cr}(a, b, c, f)= t_1 \cdot t_3, \end{aligned}$$

where $t_1 := t[c, a, b]^{-1}$, $t_2 := t[d, a, c]$, and $t_3 := t[f, a, c]$. Consequently, as all $t_i \in \mathrm{Im}\mathbb {H}$, we have

$$\begin{aligned}{}[r, v] = [\langle t_1, t_2\rangle , -t_1 \times t_2], \quad \text {and}\quad [\lambda r, \mu v] = [\langle t_1, t_3\rangle , -t_1 \times t_3]. \end{aligned}$$

Since all $t_1, t_2, t_3$ are orthogonal to *v* and therefore linearly dependent, we can express $t_3$ in the form $t_3 = \alpha t_1 + \beta t_2$. The two vectors $t_1$ and $t_2$ are linearly independent since otherwise $t_1 \times t_2$ would be zero implying $\mathrm{cr}(a, b, c, d) = [r, v] = [-\langle t_1, t_2\rangle , 0] \in \mathbb {R}$ which is a contradiction to the four points *a*, *b*, *c*, *d* not being concyclic.

After inserting $t_3 = \alpha t_1 + \beta t_2$ into the above equations we obtain

$$\begin{aligned}&\lambda \langle t_1, t_2\rangle = \lambda r = \langle t_1, t_3\rangle = \alpha \langle t_1, t_1\rangle + \beta \langle t_1, t_2\rangle , \\&\mu t_1 \times t_2 = -\mu v = t_1 \times t_3 = \alpha t_1 \times t_1 + \beta t_1 \times t_2. \end{aligned}$$

Consequently, $\beta = \mu$ and $\alpha = (\lambda - \mu ) \langle t_1, t_2\rangle /|t_1|^2$, which determines $t_3$ uniquely. From the definition of $t_3 = t[f, a, c] = (f - a)^{-1} + (a - c)^{-1}$, we then immediately get

$$\begin{aligned} f = (t_3 - (a - c)^{-1})^{-1} + a \in \mathrm{Im}\mathbb {H}. \end{aligned}$$

Furthermore, the circumsphere of *a*, *b*, *c*, *f* is the same as the circumsphere of *a*, *b*, *c*, *d* since both pass through *a*, *b*, *c* and both have parallel normal vectors ($\mu v$ and *v*, resp.) at *a*, and there is only one such sphere. $\square$

### Point-insertion-rule in $\mathbb {H}$

Let us now consider the analogous construction of (3) by inserting a new point to given four points $a, b, c, d \in \mathrm{Im}\mathbb {H}$ in three dimensional space. The quaternionic square root is not uniquely defined in our formulation (see Sec. 2.1) for negative real numbers. So we must exclude that case in the following which is not a significant restriction as this case (i.e., $\mathrm{cr}(c, a, b, d) \in \mathbb {R}_{< 0})$ corresponds to a concyclic quadrilateral *a*, *b*, *c*, *d* with *a* separated from *d* by *b* and *c* on the circumcircle. We exclude such “zigzag” quadrilaterals in the following and consider them as discrete singularities of our polygons.

The quaternionic formula analogous to (3) reads:

$$\begin{aligned} f(a, b, c, d) := \big ( (b - a) (c - a)^{-1} \sqrt{\mathrm{cr}(c, a, b, d)} + 1 \big )^{-1} \cdot \big ( (b - a) (c - a)^{-1} \sqrt{\mathrm{cr}(c, a, b, d)} c + b \big ). \end{aligned}$$

The notation of this formula is less flexible than in the complex case due to the noncommutativity of $\mathbb {H}$. As it will turn out, *f*(*a*, *b*, *c*, *d*) is purely imaginary and thus in three space, but note that a priori *f* is a quaternion. In analogy to Lemma 1*f* is also the solution to a cross-ratio equation:

#### **Lemma 11**

*The newly inserted point* *f*(*a*, *b*, *c*, *d*) *fulfills*

$$\begin{aligned} \mathrm{cr}(c, a, b, f(a, b, c, d)) = -\sqrt{\mathrm{cr}(c, a, b, d)}. \end{aligned}$$

#### **Corollary 4**

*f* *is a point in three dimensional space, i.e.,* $f \in \mathrm{Im}\mathbb {H}$. *Even more,* *f* *lies on the circumsphere of* *a*, *b*, *c*, *d*.

#### *Proof*

The square root of a quaternion $q = [r, v]$ (see Sec. 2.1) is a quaternion with imaginary part parallel to *v*, i.e., parallel to the imaginary part of *q*. Consequently, Proposition 2 implies that *f* is in $\mathrm{Im}\mathbb {H}$ and in particular on the circumsphere of *a*, *b*, *c*, *d*. $\square$

### Curvature for discrete space curves

In this section we will relate the curvature and curvature circle of discrete curves in three dimensional space to the planar case (Sec. 3.2). But first let us recall some properties of smooth curves $s: \mathbb {R}\rightarrow \mathbb {R}^3$.

Consider a sequence of four points on the curve *s* which converge to one point *s*(0). At any time the four points are assumed to uniquely determine a sphere. Consequently, as the four points converge to one point the sequence of spheres defined that way converges to the so called osculating sphere (see e.g., [7]). The *osculating sphere* passes through *s*(0) and has its center at

15 $$\begin{aligned} s(0) + \frac{1}{\kappa } N + \frac{\kappa '}{\kappa ^2 \tau } B, \end{aligned}$$

where *N* is the unit normal vector, *B* the binormal unit vector, $\kappa$ the curvature, and $\tau$ the torsion of the curve. The curvature circle at *s*(0) is the intersection of the osculating plane with the osculating sphere and thus lies on the osculating sphere.

#### **Lemma 12**

*The osculating sphere has contact of order* $\ge 3$ *with the curve* *s* *which implies that there is a curve* ${\hat{s}}$ *on the osculating sphere such that,*

$$\begin{aligned} s(0) = {\hat{s}}(0), \quad s'(0) = {\hat{s}}'(0), \quad s''(0) = {\hat{s}}''(0), \quad s'''(0) = {\hat{s}}'''(0), \end{aligned}$$

This immediately implies the following lemma.

#### **Lemma 13**

*The curvature and the curvature circle of a space curve* *s*(*u*) *at* $u = 0$ *is the same as the curvature and the curvature circle of* ${\hat{s}}$ *on the osculating sphere at* $u = 0$.

Any Möbius transformation that maps the osculating sphere to a plane also transforms the curvature circle to that plane.

Let us now define a curvature circle for discrete space curves. So let us start with a discrete curve $\gamma : \mathbb {Z}\rightarrow \mathbb {R}^3$ and set $a = \gamma _{i - 1}, b = \gamma _{i}, c = \gamma _{i + 1}, d = \gamma _{i + 2}.$ In analogy to Theorem 1 we define

$$\begin{aligned} p_{ab} = f(d, a, b, c),\ p_{bc} = f(a, b, c, d),\ p_{cd} = f(b, c, d, a),\ p_{da} = f(c, d, a, b), \end{aligned}$$

but now for the ‘quaternionic’ *f*. Lemma 11 implies that $p_{ab}, p_{bc}, p_{cd}, p_{da}$ lie on the circumsphere of *a*, *b*, *c*, *d* which we consider as the *discrete osculating sphere*.

Let us now consider a Möbius transformation that maps the osculating sphere to the [*yz*]-plane of a Cartesian *xyz*-coordinate system. This Möbius transformation (as any Möbius transformation does) keeps the real part as well as the length of the imaginary part of the cross-ratio of four points invariant. The transformed cross-ratios have imaginary parts that are orthogonal to the circumsphere of the new points (Lemma 10). Therefore the transformed cross-ratios have imaginary parts that are parallel to the *x*-axis of the coordinate system. Consequently, the cross-ratios are complex numbers [*r*, (*x*, 0, 0)], and we arrive at the case of planar curves (Sec. 3).

So, after the Möbius transformation we can apply Theorem 1 which implies that $p_{ab},$ $p_{bc},$ $p_{cd},$ $p_{da}$ lie on a common circle ${\tilde{k}}_i$ and have a cross-ratio of $-1$. Furthermore, the inverse Möbius transformation maps the circle ${\tilde{k}}_i$ to a circle $k_i$ on the osculating sphere. And since Möbius transformations map the curvature circle of a curve to the curvature circle of the transformed curve, the following definition is sensible.

#### **Definition 4**

For a discrete space curve $\gamma : \mathbb {Z}\rightarrow \mathbb {R}^3$ we call the circle $k_i$ *(discrete) curvature circle* and the inverse of its radius *curvature* $\kappa _i$ *at the edge* $\gamma _{i}\gamma _{i + 1}$. For an illustration see Fig. 5 (right).

#### **Theorem 4**

*Let* $s: \mathbb {R}\rightarrow \mathbb {R}^3$ *be a sufficiently smooth planar curve and let* $u, \varepsilon \in \mathbb {R}$. *Further let* $\gamma : \mathbb {Z}\rightarrow \mathbb {R}^3$ *be the discrete curve* $\gamma _k = \gamma (k) = s(u + (2 k - 1)\varepsilon )$. *All the approximation results from Theorem* 2*apply to space curves in* $\mathbb {R}^3$.

#### *Proof*

At first we convince ourselves that it is sufficient to replace the curve *s* by the curve ${\hat{s}}$ on the osculating sphere (Lemma 12). Thus instead of $\gamma _k$ we use ${\hat{\gamma }}_k = {\hat{s}}(u + (2 k - 1)\varepsilon )$ for the computation of the discrete curvature circle. We have ${\hat{\gamma }}_k = \gamma _k + \mathrm{O}(\varepsilon ^4)$, and therefore

$$\begin{aligned} f({\hat{\gamma }}_{i - 1}, {\hat{\gamma }}_i, {\hat{\gamma }}_{i + 1}, \hat{\gamma }_{i + 2}) = f(\gamma _{i - 1}, \gamma _i, \gamma _{i + 1}, \gamma _{i + 2}) + \mathrm{O}(\varepsilon ^4), \end{aligned}$$

i.e., the four points for which we construct the discrete curvature circle are $\mathrm{O}(\varepsilon ^4)$-close to the points on the actual discrete curvature circle. Hence, the center of the replacing curvature circle is also $\mathrm{O}(\varepsilon ^4)$-close to the actual circle since the center of the circumcircle of a triangle $a, b, c \in \mathbb {R}^3$ is

$$\begin{aligned} \frac{(\Vert a - c\Vert ^2 (b - c) - \Vert b - c\Vert ^2 (a - c)) \times ((a - c) \times (b - c))}{2 \Vert (a - c) \times (b - c)\Vert ^2} + c. \end{aligned}$$

Now we know that it is sufficient to show the 3-space version of Theorem 2 for ${\hat{s}}$ instead of *s*. After a stereographic projection from the osculating sphere to the complex plane we arrive at the case of planar curves (Sec. 3) for which Theorem 2 holds. The only thing left to prove is that the stereographic projection does not change the approximation order of the center of the curvature circle.

Let *m*, *r* denote the center and radius of the smooth curvature circle of the planar curve, and let $m_0(\varepsilon ), r_0(\varepsilon )$ denote the curvature circle of $\gamma$. From Theorem 2 we know that

$$\begin{aligned} m = m_0(\varepsilon ) + \mathrm{O}(\varepsilon ^2) \quad \text {and}\quad r = r_0(\varepsilon ^2) + \mathrm{O}(\varepsilon ^2). \end{aligned}$$

After mapping a circle in $\mathbb {C}$ with center $m = m_1 + i m_2$ and radius *r* stereographically to the sphere we obtain

$$\begin{aligned} \phi (m, r) := \frac{1}{r^2 - 2 r^2 (|m|^2 - 1) + (|m|^2 + 1)^2} \left( \begin{array}{c} 2 m_1 (1 - r^2 + |m|^2) \\ 2 m_2 (1 - r^2 + |m|^2) \\ (r^2 - 1 - |m|^2) (r^2 + 1 - |m|^2) \end{array} \right) \end{aligned}$$

for the new center. Therefore

$$\begin{aligned} \phi (m_0, r_0) = \phi (m, r) + \mathrm{O}(\varepsilon ^2), \end{aligned}$$

i.e., the centers are $\mathrm{O}(\varepsilon ^2)$-close. $\square$

## Torsion

We define the torsion for discrete curves where the cross-ratio plays a role. However, we first have to consider the right formulation of the torsion of smooth curves.

### Torsion for smooth curves

Let us reformulate the common notation of the torsion $\tau$ (see Eq. (1)):

$$\begin{aligned} \tau = -\frac{\langle s' \times s'', s'''\rangle }{\Vert s' \times s''\Vert ^2} = -\frac{\det (s', s'', s''')}{\Vert s' \times s''\Vert ^2} = -\frac{\det (s''', s', s'')}{\Vert s' \times s''\Vert ^2} = \frac{\langle s' \times s''', s''\rangle }{\Vert s' \times s''\Vert ^2} = \frac{\langle s' \times s''', s''\rangle }{\kappa ^2 \Vert s'\Vert ^6}. \end{aligned}$$

The normal unit vector *N* is the cross product of the binormal unit vector *B* and the tangent unit vector *T* and therefore reads

$$\begin{aligned} N = B \times T = \frac{s' \times s''}{\Vert s' \times s''\Vert } \times \frac{s'}{\Vert s'\Vert } = \frac{s'' \langle s' ,s' \rangle - s' \langle s', s''\rangle }{\Vert s'\Vert \Vert s' \times s''\Vert } = \frac{1}{\Vert s'\Vert ^2 \kappa } s'' -\frac{\langle s', s'' \rangle }{\Vert s'\Vert \Vert s' \times s''\Vert } s'. \end{aligned}$$

Consequently,

16 $$\begin{aligned} \tau = \frac{\langle s' \times s''', s''\rangle }{\kappa ^2 \Vert s'\Vert ^6} = \frac{\langle s' \times s''', \Vert s'\Vert ^2 \kappa N\rangle }{\kappa ^2 \Vert s'\Vert ^6} = \frac{\langle s' \times s''', N\rangle }{\kappa \Vert s'\Vert ^4}. \end{aligned}$$

We will come back to such formulation of $\tau$ in the proof of Theorem 5.

### Discrete Frenet frame

**Fig. 6:**
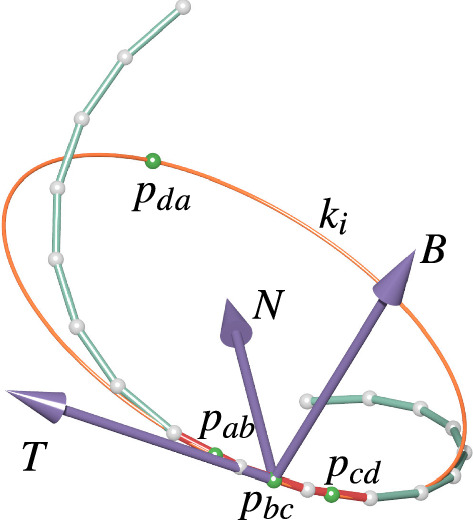
A discrete space curve with discrete curvature circle $k_i$. The discrete tangent vector *T* of the curve is defined to be the tangent vector of the curvature circle at $p_{bc}$. The discrete normal vector *N* lies in the plane of the circle and orthogonal to *T* and the binormal vector *B* is orthogonal to both

There is a natural way to define a discrete Frenet frame in our setting. Theorem 2 implies that $p_{bc} = p_{\gamma _{i} \gamma _{i + 1}}$ is a good discrete candidate for a point where the curvature circle should be in tangential contact with the curve as $p_{bc}$ is a third-order approximation of *s*(*u*). Thus it is sensible to choose the discrete unit tangent vector $T_i$ to be in tangential contact with the curvature circle at $p_{bc}$. It is therefore equally natural to define the normal unit vector $N_i$ to be the normal of the curvature circle at $p_{bc}$. Consequently, the binormal vector $B_i$ should be orthogonal to $N_i$ and $T_i$ (see Fig. 6).

#### **Lemma 14**

*Let* $s: \mathbb {R}\rightarrow \mathbb {R}^3$ *be a sufficiently smooth curve and let* $u, \varepsilon \in \mathbb {R}$. *Further let* $\gamma : \mathbb {Z}\rightarrow \mathbb {C}$ *be the discrete curve* $\gamma _k = \gamma (k) = s(u + (2 k - 1)\varepsilon )$. *Then the discrete unit normal* $N_i$ *is a second-order approximation of the smooth normal* *N*, *i.e.,*

$$\begin{aligned} N_i = N + \mathrm{O}(\varepsilon ^2). \end{aligned}$$

#### *Proof*

$$\begin{aligned} N_i = \frac{p_{bc} - m_0}{\Vert p_{bc} - m_0\Vert } \overset{(*)}{=} \frac{s + \mathrm{O}(\varepsilon ^3) - m + \mathrm{O}(\varepsilon ^2)}{\Vert s + \mathrm{O}(\varepsilon ^3) - m + \mathrm{O}(\varepsilon ^2)\Vert } = \frac{s - m}{\Vert s - m\Vert } + \mathrm{O}(\varepsilon ^2) = N + \mathrm{O}(\varepsilon ^2), \end{aligned}$$

where we used Theorem 4 at $(*)$. $\square$

#### **Lemma 15**

*With the same assumptions as in Lemma* 14*we obtain*

$$\begin{aligned} T_i = T + \mathrm{O}(\varepsilon ^2). \end{aligned}$$

#### *Proof*

Theorem 3 implies $T_i = T + \mathrm{O}(\varepsilon ^2)$ for the planar case. What remains to verify is that a Möbius transformation does not change this order.

Any vector *v* attached at a point *p* can be represented as the derivative of a straight line:

$$\begin{aligned}{}[p + t v]_{t = 0}. \end{aligned}$$

Consequently, an inversion maps that vector to

$$\begin{aligned} \left[ \frac{\mathrm{d}}{\mathrm{d}t} \frac{p + t v}{\Vert p + t v\Vert ^2}\right] _{t = 0} = \frac{\Vert p\Vert ^2 v - 2 \langle p, v \rangle p}{\Vert p\Vert ^4}. \end{aligned}$$

In our case the vector $T_i$ is attached at point $p_{bc}$. Since $T_i = T + \mathrm{O}(\varepsilon ^2)$ and $p_{bc} = s + \mathrm{O}(\varepsilon ^3)$ for planar curves, we obtain for the tangent vector after inversion

$$\begin{aligned} \frac{\Vert p_{bc}\Vert ^2 T_i - 2 \langle p_{bc}, T_i \rangle p_{bc}}{\Vert p_{bc}\Vert ^4}&= \frac{\Vert s + \mathrm{O}(\varepsilon ^3)\Vert ^2 (T + \mathrm{O}(\varepsilon ^2)) - 2 \langle s + \mathrm{O}(\varepsilon ^3), T + \mathrm{O}(\varepsilon ^2) \rangle (s + \mathrm{O}(\varepsilon ^3))}{\Vert s + \mathrm{O}(\varepsilon ^3)\Vert ^4} \\&= \frac{\Vert s\Vert ^2 T - 2 \langle s, T\rangle s}{\Vert s\Vert ^4} + \mathrm{O}(\varepsilon ^2). \end{aligned}$$

Therefore, Möbius transformations map $\varepsilon ^2$-close vectors attached at $\varepsilon ^3$-close points to $\varepsilon ^2$-close vectors. $\square$

#### **Corollary 5**

*With the same assumptions as in Lemma* 14*the discrete Frenet frame* $(T_i, N_i, B_i)$ *is a second-order approximation of the smooth Frenet frame* (*T*, *N*, *B*).

### Torsion for discrete curves

In this section we relate the torsion of a discrete curve to the cross-ratio of four successive vertices of the curve. The real part and the length of the imaginary part of the quaternionic cross-ratio are Möbius invariant but the torsion is not; hence the definition must also include other quantities that are not Möbius invariant: curvature and length. In Theorem 5 we again use asymptotic analysis to justify our definition of the discrete torsion.

#### **Definition 5**

Let $\gamma : \mathbb {Z}\rightarrow \mathbb {R}^3 \cong \mathrm{Im}\mathbb {H}$ be a discrete curve, let $\kappa _i$ be the discrete curvature at the edge $\gamma _{i} \gamma _{i + 1}$, and let $N_i$ denote the discrete normal unit vector. Then, we call

$$\begin{aligned} \tau _i := - \frac{9 \langle \mathrm{Im}\mathrm{cr}(\gamma _{i - 1}, \gamma _{i}, \gamma _{i + 1}, \gamma _{i + 2}), N_i \rangle }{2 \kappa _i \Vert \gamma _{i}\! -\! \gamma _{i + 1}\Vert ^2} \end{aligned}$$

the *(discrete) torsion of* $\gamma$ *at the edge* $\gamma _{i} \gamma _{i + 1}$.

#### **Proposition 3**

*The discrete torsion vanishes for planar discrete curves.*

#### *Proof*

Planarity of the discrete curve and Lemma 10 imply that the imaginary part of the cross-ratio in the definition of the torsion is perpendicular to that plane. The normal vector $N_i$ on the other hand is contained in the plane. Therefore the two vectors are orthogonal and the discrete torsion vanishes. $\square$

#### **Theorem 5**

*Let* $s: \mathbb {R}\rightarrow \mathbb {R}^3$ *denote a sufficiently smooth curve, let* $u, \varepsilon \in \mathbb {R}$ *and let the discrete curve* $\gamma : \mathbb {Z}\rightarrow \mathbb {C}$ *with* $\gamma _k = \gamma (k) = s(u + (2 k - 1)\varepsilon )$ *be a sampling of* *s*. *Then*

$$\begin{aligned} \tau _0 = \tau + \mathrm{O}(\varepsilon ^2). \end{aligned}$$

However, before we prove this theorem we need a preparatory lemma.

#### **Lemma 16**

*Let* $s: \mathbb {R}\rightarrow \mathbb {C}$ *denote a sufficiently smooth curve, let* $u, \varepsilon \in \mathbb {R}$ *and let the discrete curve* $\gamma : \mathbb {Z}\rightarrow \mathbb {C}$ *with* $\gamma _k = \gamma (k) = s(u + (2 k - 1)\varepsilon )$ *be a sampling of* *s*. *Further let* $q_0$ *denote the cross-ratio of four consecutive vertices* $q_0 := \mathrm{cr}(\gamma _{-1}, \gamma _0, \gamma _1, \gamma _2)$. *Then*

$$\begin{aligned} \mathrm{Re}q_0&= -\frac{1}{3} - \frac{-24 \langle s', s''\rangle ^2 + 8 \Vert s'\Vert ^2 \langle s', s'''\rangle + 12 \Vert s'\Vert ^2 \Vert s''\Vert ^2}{9 \Vert s'\Vert ^4} \varepsilon ^2 + \mathrm{O}(\varepsilon ^3), \\ \mathrm{Im}q_0&= -\frac{8 \Vert s'\Vert ^2 s' \times s''' - 24 \langle s', s'' \rangle s' \times s''}{9 \Vert s'\Vert ^4} \varepsilon ^2 + \mathrm{O}(\varepsilon ^3). \end{aligned}$$

#### *Proof*

We compute

$$\begin{aligned} \mathrm{cr}(\gamma _{-1}, \gamma _0, \gamma _1, \gamma _2) = (\gamma _{-1} - \gamma _0) (\gamma _0 - \gamma _1)^{-1} (\gamma _1 - \gamma _2) (\gamma _2 - \gamma _{-1})^{-1} \end{aligned}$$

by first expressing each factor in terms of its Taylor expansion:

$$\begin{aligned} \gamma _{-1} - \gamma _0&= -2 s' \varepsilon + 4 s'' \varepsilon ^2 - \frac{13 s'''}{3} \varepsilon ^3 + \mathrm{O}(\varepsilon ^4), \\ \gamma _1 - \gamma _2&= -2 s' \varepsilon - 4 s'' \varepsilon ^2 - \frac{13 s'''}{3} \varepsilon ^3 + \mathrm{O}(\varepsilon ^4), \end{aligned}$$

and now the inverted factors

$$\begin{aligned} (\gamma _0 - \gamma _1)^{-1}&= \Big ( -2 s' \varepsilon - \frac{s'''}{3} \varepsilon ^3 + \mathrm{O}(\varepsilon ^4) \Big )^{-1} = \frac{2 s' \varepsilon + \frac{s'''}{3} \varepsilon ^3 + \mathrm{O}(\varepsilon ^4)}{4 \Vert s'\Vert ^2 \varepsilon ^2 + \frac{4\langle s', s'''\rangle }{3} \varepsilon ^4 + \mathrm{O}(\varepsilon ^5) } \\&= \frac{s'}{2 \Vert s'\Vert ^2 \varepsilon } + \frac{\Vert s'\Vert ^2 s''' - 2 \langle s', s'''\rangle s'}{12 \Vert s'\Vert ^4} \varepsilon + \mathrm{O}(\varepsilon ^2), \end{aligned}$$

where the last equality holds since $\frac{x_0 + x_2 \varepsilon ^2 + \mathrm{O}(\varepsilon ^3)}{y_1 \varepsilon + y_3 \varepsilon ^3 + \mathrm{O}(\varepsilon ^4)} = \frac{x_0}{y_1 \varepsilon } + \frac{x_2 y_2 - x_0 y_3}{y_1^2} \varepsilon + \mathrm{O}(\varepsilon ^2)$. Analogously, we obtain

$$\begin{aligned} (\gamma _2 - \gamma _{-1})^{-1} = -\frac{s'}{6 \Vert s'\Vert ^2 \varepsilon } + \frac{-\Vert s'\Vert ^2 s''' + 2 \langle s', s'''\rangle s'}{4 \Vert s'\Vert ^4} \varepsilon + \mathrm{O}(\varepsilon ^2). \end{aligned}$$

The above four factors are all purely imaginary quaternions. Multiplying these factors together in the right order yields the proposed real and imaginary part of the cross-ratio. $\square$

So, let us now turn to our approximation result for the torsion:

#### *Proof of Theorem 5*

We show the formula at $u = 0$ and therefore $i = 0$:

$$\begin{aligned} \tau _0&= -\frac{9}{2} \frac{\langle \mathrm{Im}q_0, N_0\rangle }{\kappa _0 \Vert \gamma _0 - \gamma _1\Vert ^2} \overset{(*)}{=} -\frac{9}{2} \frac{\langle (-8 \Vert s'\Vert ^2 s' \times s''' + 24 \langle s', s''\rangle s' \times s'') \varepsilon ^2 + \mathrm{O}(\varepsilon ^3), N + \mathrm{O}(\varepsilon ^2)\rangle }{9 \Vert s'\Vert ^4 (\kappa + \mathrm{O}(\varepsilon ^2)) \Vert 2 \varepsilon s' + \mathrm{O}(\varepsilon ^3)\Vert ^2} \\&\overset{(\S )}{=} \frac{\langle \Vert s'\Vert ^2 s' \times s''' \varepsilon ^2, N \rangle + \mathrm{O}(\varepsilon ^3)}{\Vert s'\Vert ^6 \kappa \varepsilon ^2 + \mathrm{O}(\varepsilon ^2)} = \frac{\langle s' \times s''', N \rangle }{\Vert s'\Vert ^4 \kappa } + \mathrm{O}(\varepsilon ^2) \overset{(16)}{=} \tau + \mathrm{O}(\varepsilon ^2), \end{aligned}$$

where we used

$$\begin{aligned} \Vert \gamma _0 - \gamma _1\Vert&= \Vert s(-\varepsilon ) - s(\varepsilon )\Vert = \Vert s - \varepsilon s' + \frac{\varepsilon ^2}{2} + \mathrm{O}(\varepsilon ^3) - (s + \varepsilon s' + \frac{\varepsilon ^2}{2} + \mathrm{O}(\varepsilon ^3))\Vert \\&= \Vert 2 \varepsilon s' + \mathrm{O}(\varepsilon ^3)\Vert \end{aligned}$$

at $(*)$ and $\langle s' \times s'', N\rangle = 0$ at $(\S )$. $\square$

#### *Remark 2*

We have now a curvature and torsion for a discrete space curve as well as an osculating sphere and osculating circle. In the setting of smooth curves the oriented distance between the center of the osculating circle and the osculating sphere is

$$\begin{aligned} \frac{\kappa '}{\kappa ^2 \tau } \end{aligned}$$

as follows immediately from the formula for the center of the osculating sphere, Eq. (15). We can therefore define a discrete version of $\kappa '$ as that value that fulfills the equation above by replacing smooth notions by their discrete counterparts.

## Geometric properties

**Fig. 7:**
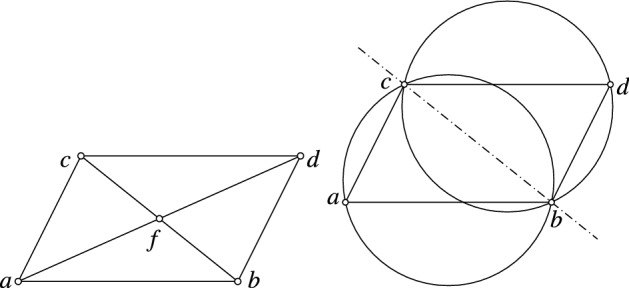
Left: Any quadrilateral *a*, *b*, *c*, *d* is Möbius equivalent to a parallelogram with $b - a = d - c$. In case of such a parallelogram we have $\mathrm{cr}(c, a, b, f) = -\sqrt{\mathrm{cr}(c, a, b, d)}$. Right: The circumcircles of *a*, *b*, *c* and *b*, *d*, *c* are congruent. One of their two bisector circles is a straight line, the diagonal *cb*

Any quadrilateral is Möbius equivalent to a parallelogram, and especially Möbius equivalent to a parallelogram *a*, *b*, *c*, *d* with $a = 0$, $b = 1$ and $b - a = d - c$. See Fig. 7 (left). Its cross-ratio is

$$\begin{aligned} \mathrm{cr}(c, a, b, d) = c^2. \end{aligned}$$

If *f* denotes the intersection point of the diagonals then we obtain

$$\begin{aligned} \mathrm{cr}(c, a, b, f) = -c, \end{aligned}$$

and therefore

$$\begin{aligned} \mathrm{cr}(c, a, b, f) = - \sqrt{\mathrm{cr}(c, a, b, d)}. \end{aligned}$$

This together with Lemma 1 immediately implies the following Lemma.

### **Lemma 17**

*Let* *a*, *b*, *c*, *d* *be a parallelogram with* $b - a = d - c$. *Then the insertion point* *f*(*a*, *b*, *c*, *d*) *corresponds to the intersection point of the diagonals.*

Furthermore, in the case of a parallelogram the circumcircles of *a*, *b*, *c* and *b*, *d*, *c* are congruent. Therefore, one of their two bisector circles is the straight line containing the diagonal *bc* (see Fig. 7 right). The same holds for the other pair of circumcircles *a*, *b*, *d* and *a*, *d*, *c*. Since Möbius transformations do not change the intersection angles of curves we obtain the following lemma.

### **Lemma 18**

*The insertion point* *f*(*a*, *b*, *c*, *d*) *is one of the intersection points of the bisector circles of the pairs of circumcircles mentioned above.*

**Fig. 8:**
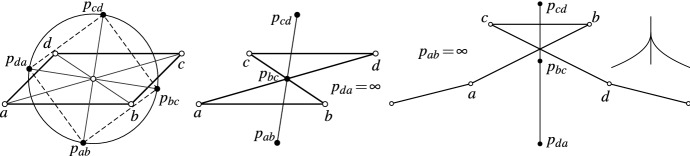
Special cases of four points together with their curvature circle: Left: The points $p_{ab}, p_{bc}, p_{cd}, p_{da}$ form a square. Center: The curvature circle degenerates to a straight line. Right: Symmetric curve with a “loop” that can be interpreted as discrete cusp of the curve. Consistently, the curvature circle degenerates to a straight line

Consequently, the discrete curvature circle can be constructed with a compass and a straight edge. In the following lemma we mention three special cases:

### **Lemma 19**

(i) Let *a*, *b*, *c*, *d* be a parallelogram with $b - a = c - d$. Then the four points $p_{ab}, p_{bc}, p_{cd}, p_{da}$ form a square (see Fig. 8 left).
(ii) Let *a*, *b*, *c*, *d* be a parallelogram with $b - a = d - c$. Then $p_{da} = \infty$ and the curvature circle degenerates to a straight line (see Fig. 8 center).
(iii) Let *a*, *b*, *c*, *d* be symmetric as in Fig. 8 (right). Then $p_{ab} = \infty$ and the curvature circle degenerates to a straight line. Thus, this arrangement of points can be seen as a discrete analogue of a cusp on a curve.

### *Proof*

ad (i): The rotational symmetry by an angle of $\pi$ of the parallelogram implies that the points $p_{ab}$ and $p_{cd}$ are opposite of the center of rotation as well as $p_{bc}$ and $p_{da}$. A quadrilateral with this property and with a cross-ratio of $-1$ (Theorem 1) must be a square.

ad (ii) and (iii): It follows from simple computations that $p_{da}$ and $p_{ab}$, respectively, vanish to $\infty$. Circles containing this point are straight lines. $\square$

## Experimental results

**Fig. 9:**
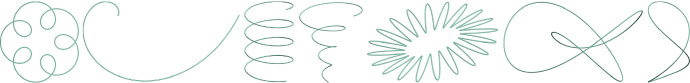
Illustration of the list of curves used for our numerical convergence verification

We conducted convergence tests which empirically verify our claims. For this, we used the following seven curves (see Fig. 9 for their depiction):

(i) The epitrochoid $c_1(t) = (6 \cos (t) - 3 \cos (6t), 6 \sin (t) - 3 \sin (6t))$ (the curve is planar).
(ii) A planar logarithmic spiral $c_2(t) = e^{at} (\cos (t), \sin (t))$, where we use $a = 0.5$.
(iii) A helix $c_3(t) = (\cos (at), \sin (at), b t)$ where we use $a=4$ and $b=0.5$.
(iv) A helical spiral $c_4(t) = (e^{at} \cos (4t), e^{at} (\sin (4t), bt)$ where we use $a=0.4$ and $b=4$.
(v) A toroidal “coil” $c_5(t) = ((a + \sin (bt)) \cos (t), (a + \sin (bt)) \sin (t), \cos (bt))$ where we use $a=2.5$ and $b=20$.
(vi) The trefoil knot $c_6(t) = (\sin (t) + 2 \sin (2t), \cos (t) - 2 \cos (2t), -\sin (3t))$.
(vii) Viviani’s curve [8] $c_7(t) = (a (1 + \cos (2t)), a \sin (2t), 2 a \sin (t))$ with $a=5$.

For all examples, we used $t \in [0, 2\pi ]$. For simplicity, we assumed all curves are open, and disregarded boundaries, that is, we do not compute edge midpoint and consequent quantities for edges adjacent to boundary vertices. The curves are not assumed to be arc-length parametrized.

**Fig. 10:**
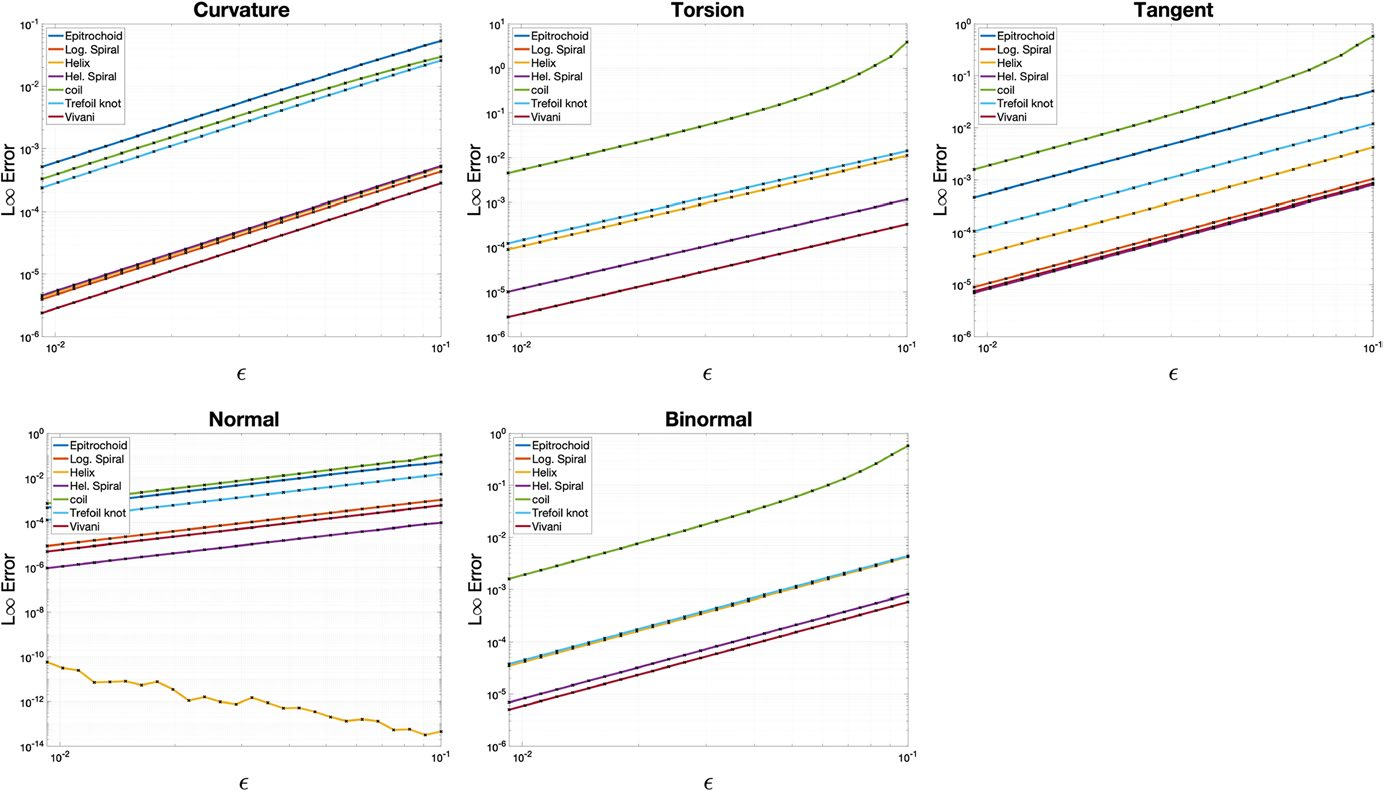
$l^{\infty }$ errors versus sampling step $\varepsilon$

For any given resolution step $\varepsilon$, we created a discrete curve by sampling every curve $c_i(t)$, as explained in Theorem 4. Then, we compute the discrete curvature $\kappa$, the discrete torsion $\tau$, and the discrete Frenet frame $\left\{ T,N,B\right\}$ for every midedge point. We measure the approximation error to the corresponding quantities of the smooth curve at the sampled points by the $l^{\infty }$ norm. This produces the maximum absolute deviation of every discrete quantity from the ground truth. In case of vector quantities (like the Frenet frame), we do so per component. We use $\varepsilon = 0.1 \times 1.1^l$, where $l \in \mathbb {N}$ runs between 0 and $-15$ in steps of $-1$, which creates gradual refinement. To measure convergence rate, we perform linear regression on the logarithmic scale of $\varepsilon$ vs. $l^{\infty }$ error per curve. The graphs of errors can be seen in Fig. 10, and the convergence rates are in Table 1. It is evident that we are able to reproduce the quadratic convergence rates that we prove in this paper. Note that we do not measure torsion for $c_1(t)$ and $c_2(t)$ as they are planar. Another outlier is the normal error for $c_3(t)$ which is already initially very low (due to the high regularity of the helix), and thus we only see the effect of numerical noise.

**Table 1:** Error convergence rates with refinement. Note that there is no torsion or non-trivial binormal for the planar curves $c_1(t)$ and $c_2(t)$

Curve	$\kappa$	$\tau$	*T*	*N*	*B*
(i)	1.9589	–	1.9858	1.9858	–
(ii)	1.9745	–	2.0005	2.0005	–
(iii)	2.0010	2.0212	2.0122	–	2.0122
(iv)	1.9947	1.9934	2.0003	1.9742	2.0002
(v)	1.9096	2.5707	2.3352	2.0647	2.3414
(vi)	1.9772	1.9936	1.9888	1.9864	1.9980
(vii)	1.9986	2.0102	2.0000	1.9996	2.0002
